# Plastic adjustments in xylem vessel traits to drought events in three *Cedrela* species from Peruvian Tropical Andean forests

**DOI:** 10.1038/s41598-022-25645-w

**Published:** 2022-12-07

**Authors:** Ernesto C. Rodríguez-Ramírez, M. Eugenia Ferrero, Ingrith Acevedo-Vega, Doris B. Crispin-DelaCruz, Ginette Ticse-Otarola, Edilson J. Requena-Rojas

**Affiliations:** 1 Laboratorio de Dendrocronología, Universidad Continental, Urbanización San Antonio, Avenida San Carlos 1980, Huancayo, Junín Peru; 2 Instituto Argentino de Nivología, Glaciología y Ciencias Ambientales (IANIGLA), CONICET-Universidad Nacional de Cuyo, Avenida Ruiz Leal S/N, Mendoza, Argentina; 3 Programa de Pós-Graduação em Ciências Florestais, Universidade Federal Rural de Pernambuco, Recife, 52171-900 Brazil; 4 Programa de Investigación de Ecología y Biodiversidad, Asociación-ANDINUS, Calle Miguel Grau 370, Sicaya, Huancayo, Junín, Peru

**Keywords:** Plant ecology, Plant stress responses, Ecology, Ecology, Environmental sciences

## Abstract

*Cedrela* species occur within the Tropical montane cloud forest (TMCF) and rainforest in North America (Mexico), Central and South America. We assessed the hypothesis that functional xylem hydraulic architecture might be influenced by specific climatic variations. We investigated the effect of climate on tree-ring width and vessel traits (diameter, vessel density, vulnerability index and hydraulic diameter) of three relict-endemic and threatened *Cedrela* species (*Cedrela fissilis*, *C*. *nebulosa* and *C*. *angustifolia*) inhabiting Peruvian Tropical Andean cloud forests. All *Cedrela* species showed a significant reduction in radial growth and adjusted vessel trait linked with temperature, precipitation, and evapotranspiration. Ring-width and vessel traits showed adaptation within *Cedrela* species, crucial to understanding a rough indication of the plant’s ability to withstand drought-induced embolism or cavitation. Our results provide evidence for hydraulic mechanisms that determine specific wood anatomical functionality to climatic variation and drought responses. Therefore, changing the frequency or intensity of future drought events might exceed the adaptive limits of TMCF tree species, resulting in a substantial reduction of hydraulic functionality in Peruvian *Cedrela* species.

## Introduction

South American Tropical montane cloud forests (TMCFs) are exceptionally diverse with rich assemblages of relict-endemic species creating hotbeds of endemism^1^. These ecosystems are distributed along an altitudinal gradient from ~ 700 to 3900 m a.s.l., from the sub-montane forests near the Amazon basin to the montane forests-puna transition^2^. Nevertheless, increasing temperatures, changing precipitation regimes and anthropogenic activities such as illegal logging, grazing and farming are threatening the conservation of these ecosystems through biodiversity loss and forest fragmentation^3,4^ which consequently affect the niche conservatism of the TMCF community^5,6^.

Drought events play a major role in forest decline and tree mortality^7,8^. Variation in vessel traits linked to tree hydraulic properties have received considerable worldwide attention in recent decades^9,10^. Tree response to drought is usually related to the morphological adaptations of vessel traits in tree rings^11^. Vessel traits (e.g., arrangement of conduits, frequency, diameter, wall thickness) is a cue to how tree species evolve as a strategy to tolerate drought-induced cavitation^12,13^. The analysis of vessel traits across time and among sites, based on exact-dated growth-rings series is a key tool to assess hydraulic adaptation response to specific climatic phenomena such as drought events. This approach complements traditional tree-ring width assessments based on reconstructions of past environmental conditions and contributes to the assessment of how extreme events impact xylem anatomy and affect tree responses to hydric stress^14^.

Specific climate and local environment fluctuations are the major evolutionary triggers in driving adaptive variation and vessel traits function in TMCF tree species^15^. Vessel traits and moisture requirements are essential to water use, growth, and responses of trees to drought^11^ which play a key role in the amount of fog, vapor plumes, drizzle, and rainfall rates^16,17^. Several tree-ring width chronologies based on *Cedrela* species have been developed in Peruvian tropical forests to describe climate-growth relationships^18,19^; however, it is still unknown how *Cedrela* species adjust vessel characteristics in response to water deficit. In the present study, we tested the hypothesis that the vessel traits more than tree-ring width, might be influenced by specific climatic variations, reflecting a hydraulic safety trade-off among species growth. Species were expected to vary in their response because of their different wood anatomical plasticity capacities. The main objectives were to (1) develop exact-dated tree-ring chronologies for the three *Cedrela* species; (2) identify the most relevant climatic factors that influence radial growth among the *Cedrela* species; (3) evaluate the resistance, recovery, and resilience of the *Cedrela* species caused by drought events; and (4) assess the vessel traits (vessel diameter, vessel density, vulnerability index, and hydraulic diameter) on specific tree-rings developed during historical drought events and the local climate effects on vessel traits.

## Results

### Tree-ring width chronologies and drought effect

**Figure 1:**
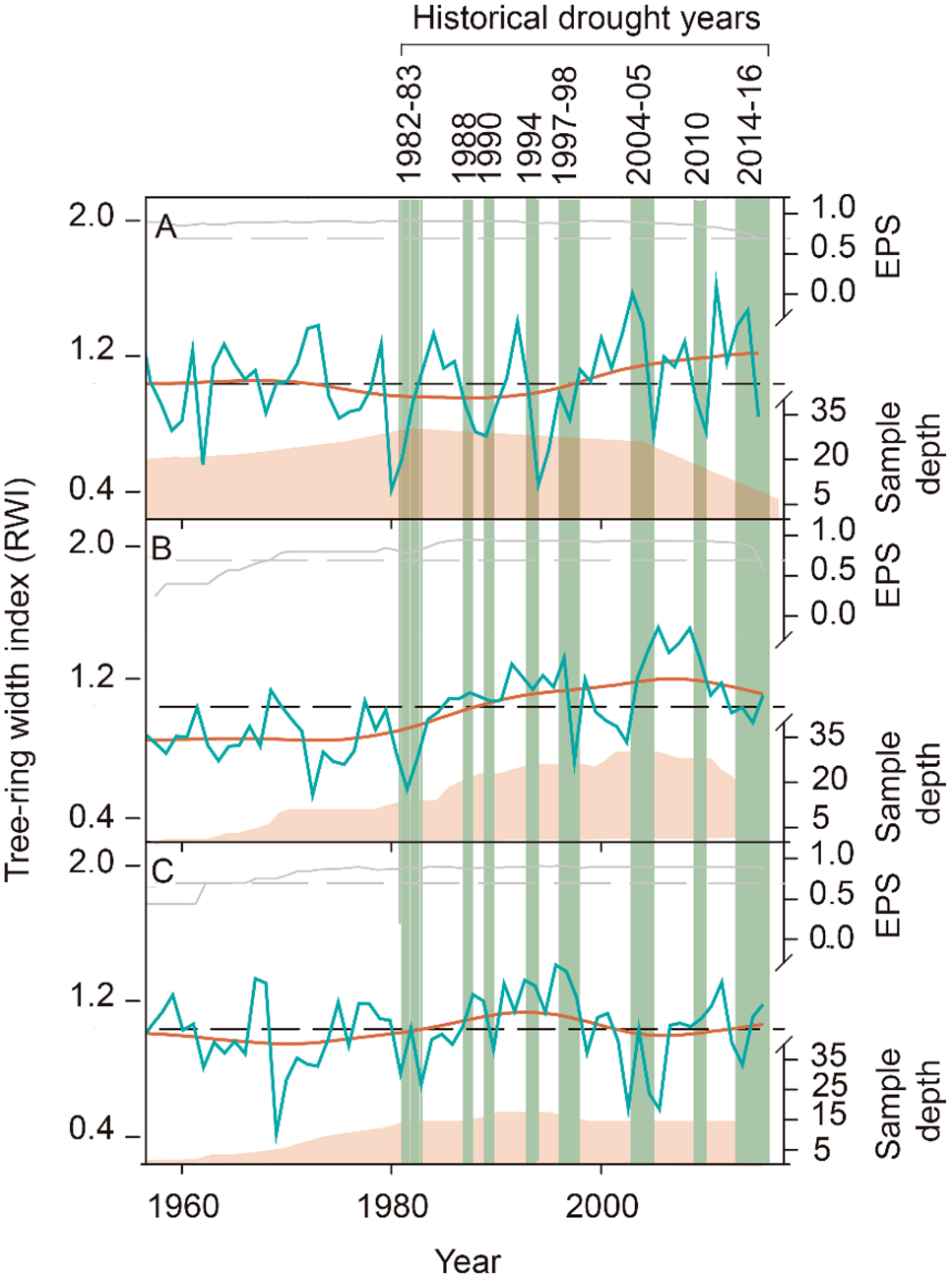
Chronologies of (**A**) *Cedrela fissili*s; (**B**) *C. nebulosa*; and (**C**) *C. angustifolia*. Each time series show a smoothed version (orange line) using a 12-year cubic spline*.* Statistical features are shown by the running Expressed Population Signal (EPS) where the horizontal dashed line shows the 0.70 EPS threshold, and the sample depth through time for each chronology. Vertical green bands indicate specific drought years.

*Cedrela* tree-rings were demarcated by a marginal parenchyma band thereby allowing vessel traits to be correctly visualized in digital images of wood cores. The cross-dated chronologies of *Cedrela fissilis* spanned 120 years and covered a period from 1896 to 2015 (Fig. 1A); for *C*. *nebulosa* we obtained a chronology of 59 years that covered the period from 1958 to 2016 (Fig. 1B), and for *C*. *angustifolia* we obtained a chronology of 62 years that covered the period from 1955 to 2016 (Fig. 1C); however, we used the chronologies from 1960 to 2016 years (Fig. 1) to assess the historical drought effect on ring-width index (RWI) and vessel traits. The expressed population signal (EPS values) of the three *Cedrela* chronologies ranged from 0.81 to 0.82, indicating the three chronologies captured a high percentage of variance of a population chronology (infinitely replicated). The mean correlation coefficient among tree-ring series (R-bar values) ranged from 0.19 to 0.42 and the inter-series correlation ranged from 0.41to 0.56 (Table S1). Both statistics demonstrate the strength and presence of common signals between width series from each chronology.

### Linking tree-ring width and climate factors

**Figure 2:**
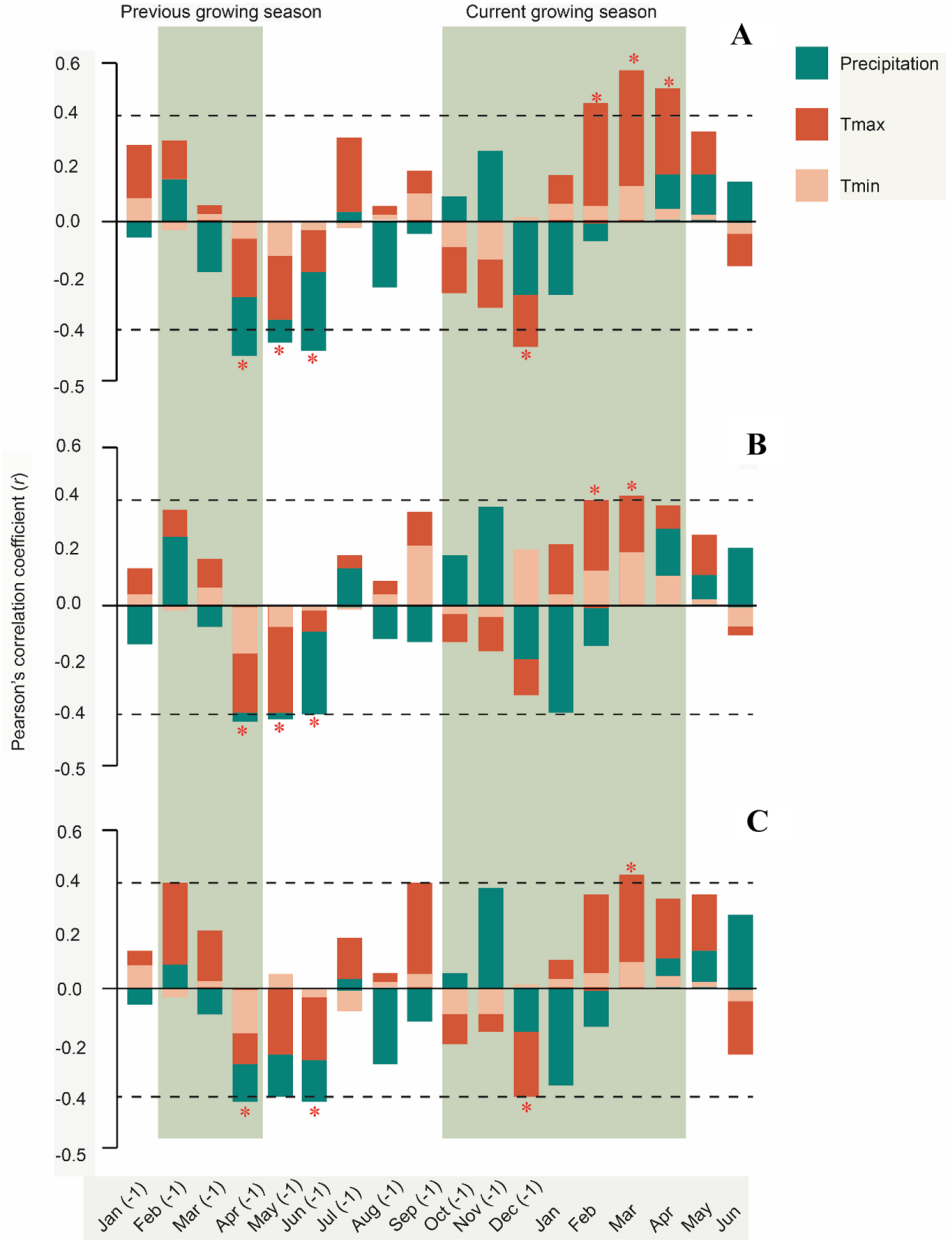
Correlations between chronologies and mean maximum temperature, mean minimum temperature and monthly precipitation from previous January (− 1) to current June in which growth started. Horizontal dashed lines indicate the significance intervals (* = *P* < 0.05) for correlation coefficients. (**A**)  *Cedrela fissilis*; (**B**) *C. nebulosa*; and (**C**) *C. angustifolia*.

Our analysis demonstrated differences in climate factors that can strongly influence the growth of *Cedrela* species. Correlations between growth rings and climate showed a significant influence of climatic variables, especially temperature (T_max_) during the growing season from October to April (Fig. 2). Precipitation (Prec) showed negative correlations in previous April (− 1) to July (− 1) months for the three chronologies. Mean maximum temperatures (T_max_) displayed consistent and positive responses with all the chronologies from February to April/May of the current growing season.

**Figure 3:**
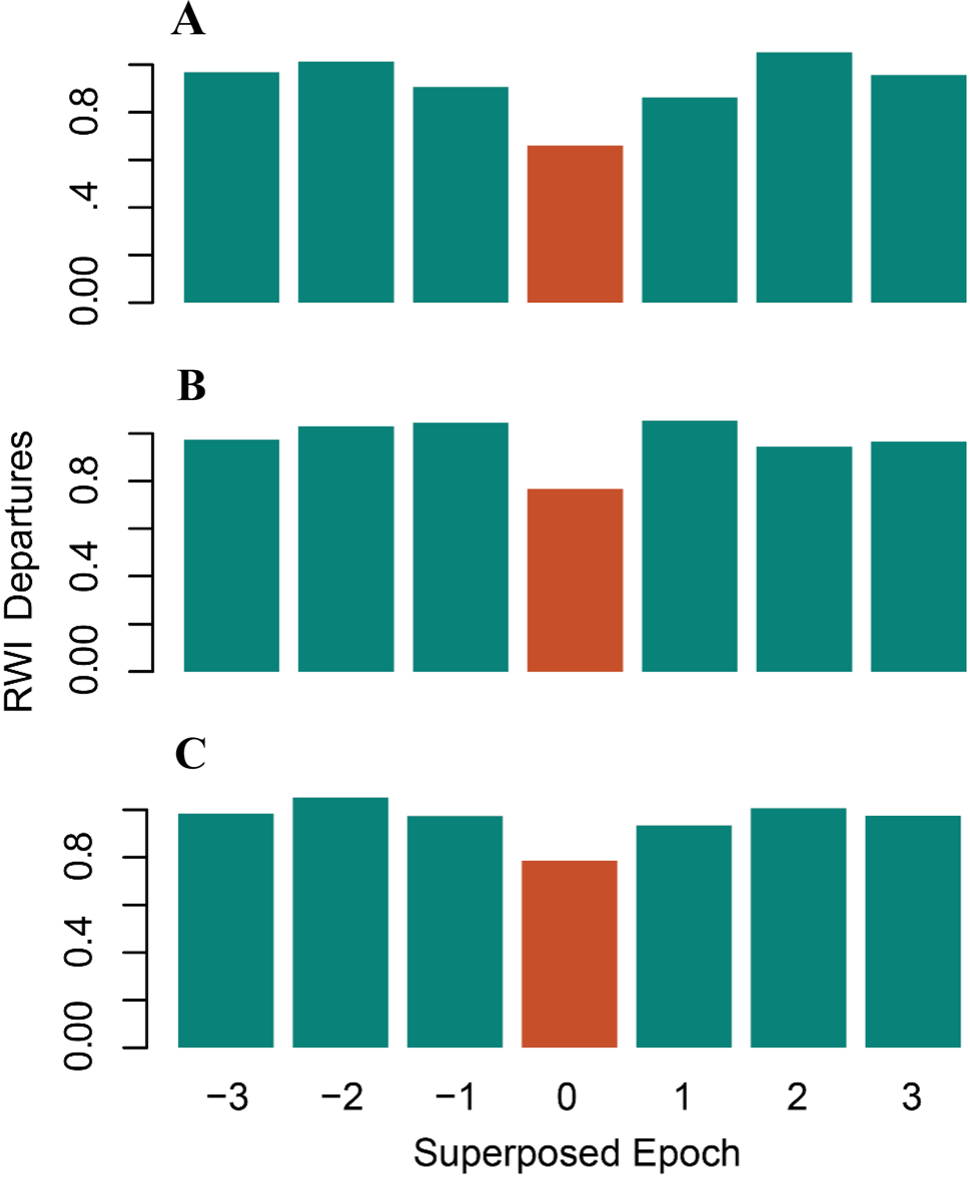
Superposed epoch analysis (SEA) comparing the RWI during specific Peruvian Andean drought events years for (**A**) *Cedrela fissilis*; (**B**) *C. nebulosa*; and (**C**) *C. angustifolia*. The X-axis represents a dataset of 7 years, from 3 years before the drought event to 3 years following it. The red bars represent the 95% confidence levels after 1000 bootstrap simulations. The common period of the three *Cedrela* species span from 1958 to 2015.

**Figure 4:**
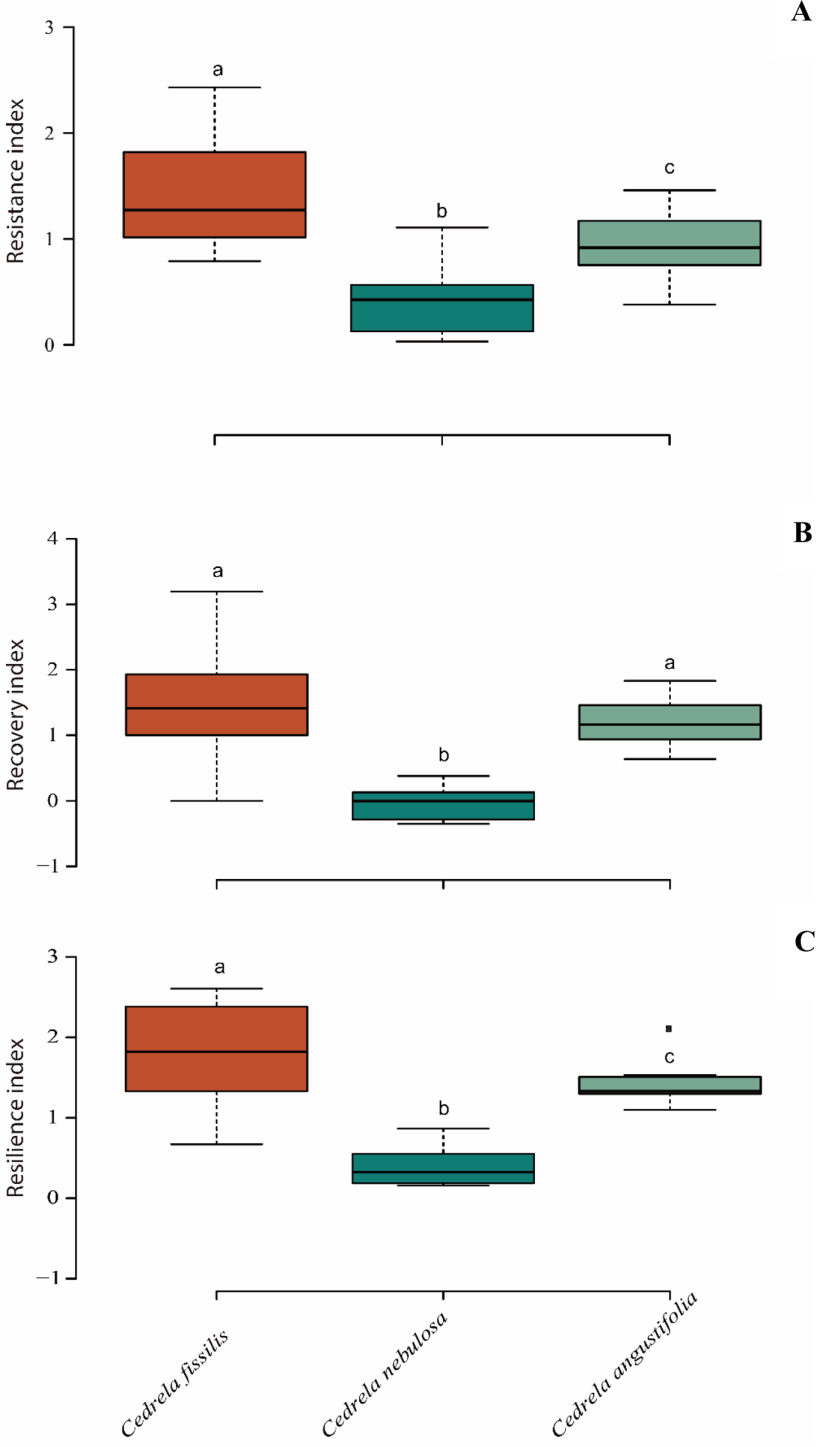
Box plots showing the variation ranges of the RWI: (**A**) Resistance index; (**B**) Recovery index; and (**C**) Resilience index among *Cedrela* species. The upper and lower limits of the boxes represent the 25th and 25th percentiles, and whiskers represent the 90th and 10th percentile. Center lines represent the medians and the solid lines within each box show statistically significant differences (*P* < 0.05). Box plots with different letters are significantly different as tested using a *post*-*hoc* Tukey test.

Additionally, the Superposed Epoch Analysis exhibited a significant reduction in the growth width for all three *Cedrela* chronologies during the drought years (Fig. 3), demonstrating a negative growth response to drought. The analyses of resistance (*Rt*) of RWI to drought detected among *Cedrela* species displayed high resistance (*Rt* = 0.5–2; Fig. 4A) after drought events. Likewise, *Cedrela fissilis* revealed a high recovery level (ability to return to pre-disturbance growth levels; *Rc* = 1.49) its tree-ring width attribute values after drought effect compared with *C. nebulosa* and *C*. *angustifolia* (*Rc* =  ~ 1.22; Fig. 4B). Finally, *C. fissilis* showed a high capacity to reach growth rates similar to those prior to a given drought event (resilience, *Rs* = 1.12) whereas *C. nebulosa* was found to be less resilient to drought compared to the other two *Cedrela* species (Fig. 4C).

### Drought effect on vessel architecture

**Figure 5:**
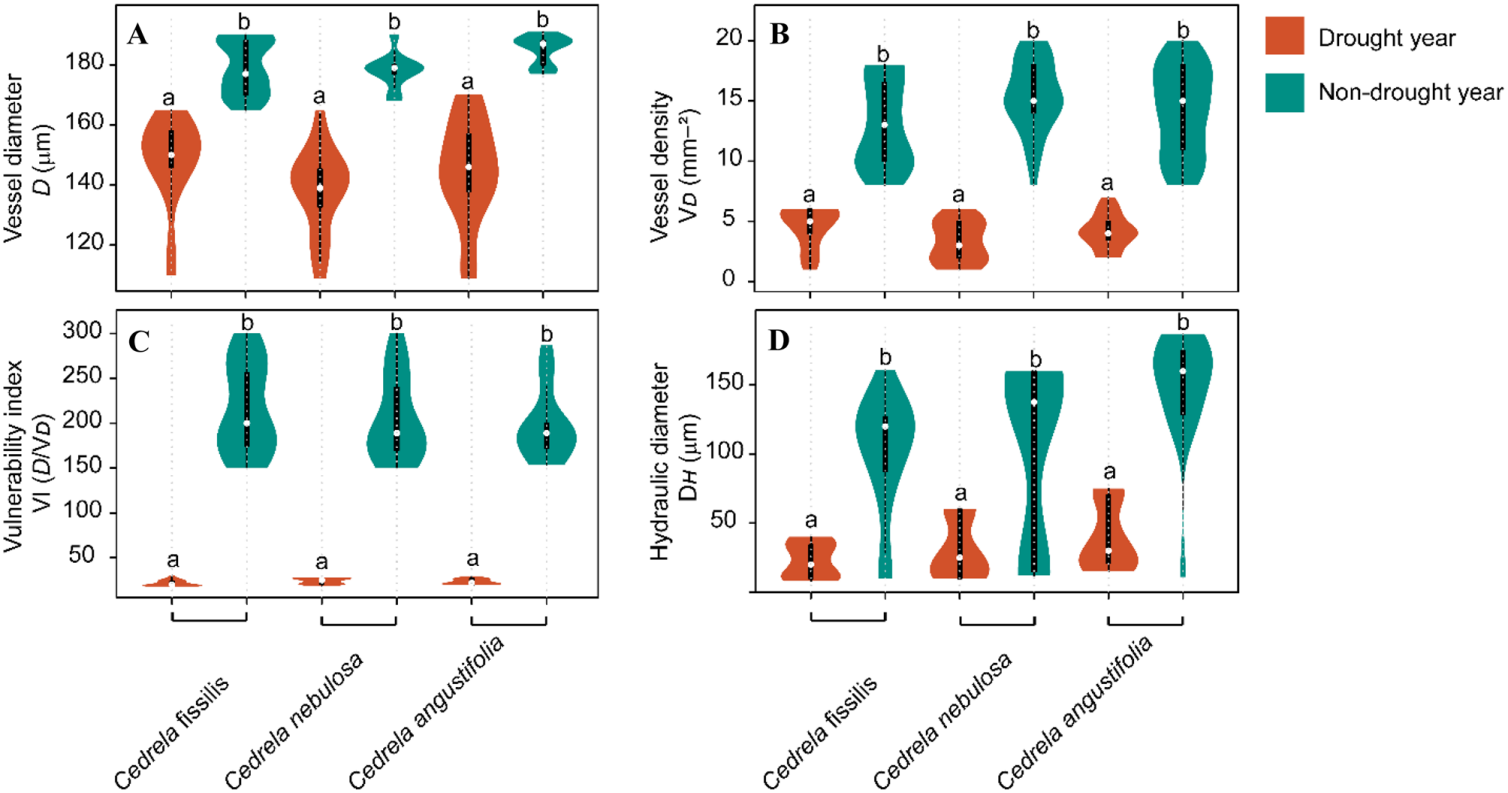
Violin plots showing differences in vessel traits measurements between drought years and non-drought years for the three *Cedrela* species. (**A**) vessel diameter; (**B**) vessel density; (**C**) vulnerability index; and (**D**) hydraulic diameter. Violin plots with different letters are significantly different as tested using a post-hoc Tukey test.

We compared the hydraulic functionality of vessels that developed during drought years (DY), and non-drought years (NDY) among *Cedrela* species using *post-hoc* Tukey tests (Fig. 5). Notably, differences between DY and NDY were mostly because of the variance among the three *Cedrela* species in vessel diameter (*D*), vessel density (V_*D*_), vulnerability index (VI) and hydraulic diameter (D_*H*_) (Fig. 5). The *D* was similar among *Cedrela* growth rings’ displaying a high plasticity during drought years (ranging from 110 to 150 μm), nevertheless, the *D* developed during non-drought years ranged from 165 to 192 (Fig. 5A). The V_D_ developed during drought years ranged from 1 to 7 mm^−2^ and 8 to 20 mm^−2^ during non-drought years (Fig. 5B). Likewise, VI was similar during drought years among *Cedrela* species (from 15 to 40 *D*/V_*D*_) and from 150 to 300 in non-drought years (Fig. 5C). The D_*H*_ values during drought years were considerably smaller (from 5 to 40 μm) than in non-drought years (from 5 to 170 μm); however, these differences were not significant, in part because of the large variance in D_*H*_ a measure of morphological adaptability recorded among species during non-drought years (Fig. 5D).

**Figure 6:**
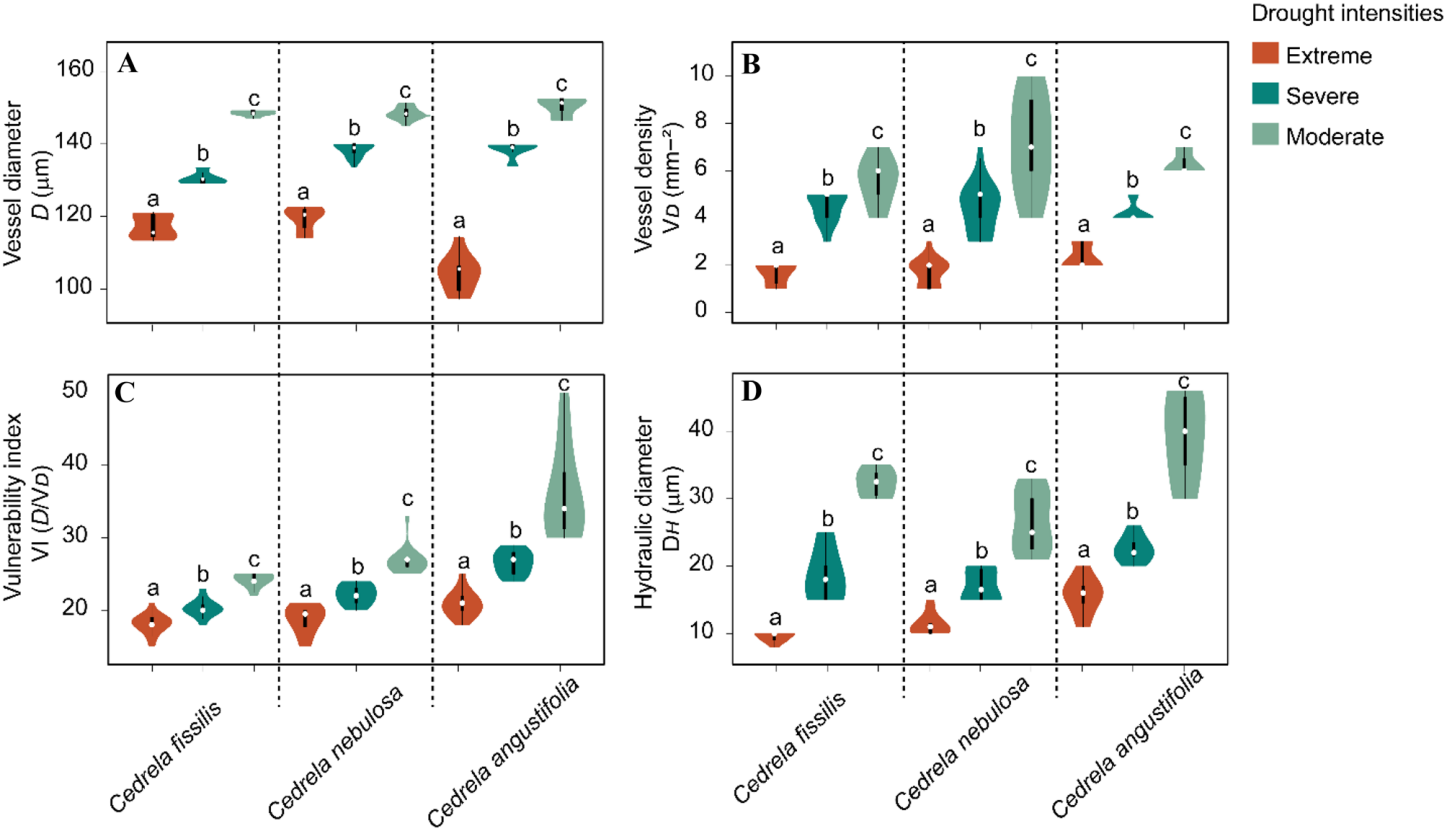
Violin plots showing vessel traits: (**A**) vessel diameter; (**B**) vessel density; (**C**) vulnerability index; and (**D**) hydraulic diameter among three drought intensities (moderate, severe, and extreme) at each *Cedrela* species. Violin plots with different letters are significantly different as tested using a post-hoc Tukey test.

We assessed the effects of drought intensities (moderate, severe, and extreme) on the four-vessel traits and found a similar response for all *Cedrela* species (Fig. 6). During drought events of extreme intensity, the *D* values were similar among *Cedrela* species (from 100 to 121 μm; Fig. 6A). In severe drought events, *D* showed a range from 129 to 140 μm, and in moderate drought events, the *D* ranged from 140 to 150 μm (Fig. 6A).

In extreme drought events, V_*D*_ ranged from 1 to 3 mm^−2^ and in severe drought events from 3 to 6 mm^−2^ for all *Cedrela* species, whereas a range from 3 to 9 mm^−2^ was found during moderate drought events (Fig. 6B). No significant differences in the V_*D*_ values were found between severe and moderate drought years in *C*. *fissilis* and *C*. *nebulosa*, but a significant difference was found for *C*. *angustifolia* (Fig. 6B). Furthermore, V_*D*_ values for each drought intensity were not significantly different among the three *Cedrela* species, except for the extreme event V_*D*_ values between *C*. *fissilis* and *C*. *angustifolia* (Fig. 6B). The VI during extreme drought events was highly plastic in *Cedrela fissilis* and *C. nebulosa* (from 15 to 25 *D*/V_*D*_) but similar values were also found in moderate drought events (from 22 to 50 *D*/V_D_) for all the *Cedrela* species (Fig. 6C). Finally, the D_*H*_ of *Cedrela fissilis* dispayed extremely narrow values during extreme drought events (from 3 to 10 μm; Fig. 6D), whereas *C. nebulosa* and *C. angustifolia* presented wider ranges (from 10 to 20 μm) during these extreme drought events. *Cedrela fissilis* showed high D_*H*_ morphological adaptability (from 15 to 22 μm) during severe drought events (Fig. 6D), like *C*. *nebulosa* and *C*. *angustifolia*, but higher during moderate drought events. The D_*H*_ values for *Cedrela fissilis* showed narrower values (from 22 to 45 μm) than *C*. *nebulosa* and *C*. *angustifolia* during the same moderate drought events (from 22 to 50 μm; Fig. 6D).

### Climate effect on xylem vessel traits

**Figure 7:**
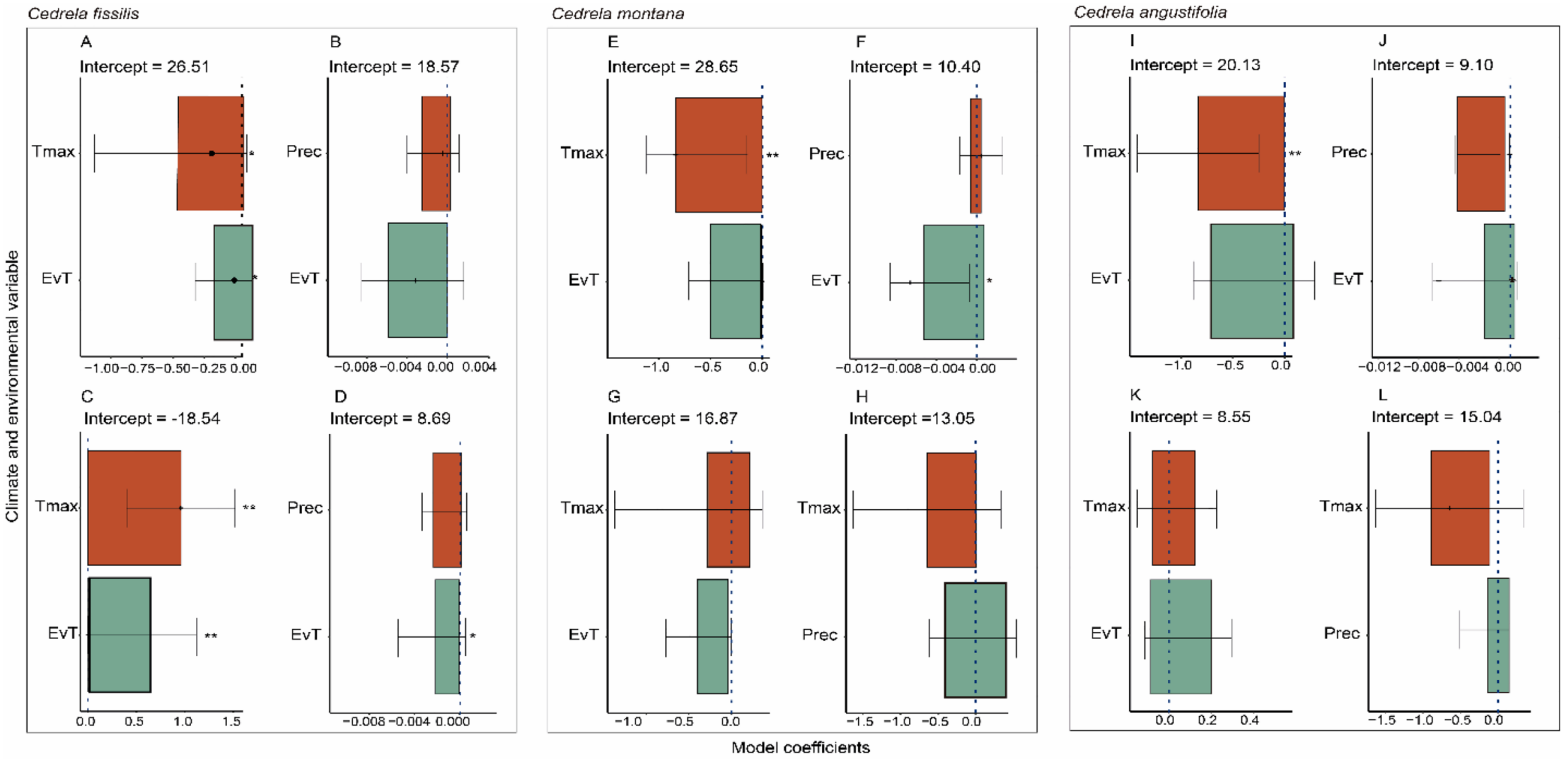
Average coefficients model of the T_max_, EvT and Prec variables that influence the vessel traits in three *Cedrela* species: (**A**,**E**,**I**) vessel diameter; (**B**,**F**,**J**) vessel density; (**C**,**G**,**K**) vulnerability index; and (**D**,**H**,**L**) hydraulic diameter. Dotted line indicates the data tendency. The asterisks show significant differences (*P < 0.05; **P < 0.01).

For all *Cedrela* species, we detected a significant positive influence of T_max_ on *D* values [*C*. *fissilis* (*β* = 0.545, *P* = 0.040; Fig. 7A); *C. nebulosa *(*β* = 0.837, *P* = 0.017; Fig. 7E); and *C*. *angustifolia* (*β* = 0.747, *P* = 0.025; Fig. 7J)]. Nevertheless, only in *C*. *fissilis* did EvT have a positive influence on *D* (*β* = 0.005, *P* = 0.018; Fig. 7B) and a negative influence on the vulnerability index (VI) values (*β* = − 0.960, *P* = 0.006); likewise, the EvT negatively affected the VI of *C*. *fissilis* (*β* = − 0.005, *P* = 0.080; Fig. 7D) (Table S2).

## Discussion

Although there has been a long-standing recognition of the importance of climate variation on tree-ring width variation for TMCF tree species^20^, the assessment of how drought influences vessel traits are relatively new. How climate triggers resilience in vessel traits may increase our understanding of how TMCF tree species will respond to future climate change^15^. Our findings demonstrate that Peruvian Andean *Cedrela* species display specific wood anatomical adjustments more so than growth-ring width and are suitable variables to explore the ecological role of tree growth in acclimation of *Cedrela* species to degraded environments^21–23^. In this context, the present study provides relevant new data on the responses of *Cedrela* species to regional drought fluctuations. We state that wood morphological adaptability is crucial to identifying the plasticity of TMCF trees to climate and micro-environmental variation (e.g., pH, high moisture and closed canopy)^15^. Previous studies on other *Cedrela* species produced similar results on *Cedrela* growth^24–26^. In this study, *Cedrela* species RWIs were correlated to T_max_ through current March to April and negatively influenced growth rates in December (− 1); moreover, T_min_ was not statistically significant with annual growth. According to Layme-Huaman et al.^19^ for *C*. *nebulosa* the maximum growth rate occurs in current March but rapidly decreases in current May. Potentially, specific environmental factors such as moisture effect (such as vapor plumes, fog, and drizzle^27^) or canopy cover could play a key role during specific drought events, subsequently displayed in narrow rings. The *Cedrela* growth observed was also negatively correlated with precipitation from April to June influencing the annual growth ring. A similar effect was detected in the Equatorial *Cedrela montana*^28^, Bolivian *C*. *fissilis* and *C*. *angustifolia*^24^, and Peruvian *C*. *nebulosa*^19^. These results could be linked to the end of the growth period in the Peruvian Andean *Cedrela* species caused by a dry-cold season and/or specific microenvironmental fluctuations such as air temperature, mist, fog and/or drizzle oscillations that influence cell division during the growth season^29^. Dendroecological and wood anatomical tools have allowed us to assess and compare specific historical drought effects on growth rings and vessel traits in *Cedrela* species back to 1982 through 2016. The Superposed Epoch Analysis showed additional evidence of the sensitivity of *Cedrela* species to drought events. During the occurrence of drought periods, particularly severe and extreme events, we observed that growth was, on average, significantly lower than normal, indicating synchronization in the growth response of the three independent chronologies during these specific drought events. The three growth sensitivity indicators (resistance, recovery, and resilience) showed specific drought sensitivity within each *Cedrela* species. During the occurrence of drought periods, we detected that the resistance (*Rt*), which quantifies the growth effect during drought events, for *Cedrela nebulosa* showed lower values even though it is located near creeks. Hence, during drought years, TMCF soil water deficits begin sooner and become more severely felt by conspecific trees in denser stands, reducing growth further^30^. Nevertheless, specific *Cedrela* species directly influence tree-ring functional and structural features that allow them to return to pre-disturbance growth rates^31^. In addition, drought effects are not always more severe in denser stands because of greater environmental stress^15^. In contrast, recovery (*Rc*), resilience (*Rs*), and resistance (*Rt*) were higher in *Cedrela fissilis* low-density stands, demonstrating that this species can thrive even in marginal sites of its range under sub-optimal soil conditions in contrast to *C. nebulosa*, which grows under high moisture conditions^19^. Thus, in theory, a high resistance index could be produced simply by poor growth before the drought events, avoiding a sharp decrease in growth during the hydric stress, and a corresponding argument holds for the resistance index (Fig. 4)^30^. Nonetheless, the growth sensitivity indicators assessment approach used here and in other studies has proven to be useful for assessing tree growth during drought events in comparisons within or between species^32^.

Our results suggest that diffuse and semi-ring porous *Cedrela* species may develop smaller vessels in markedly drought periods, suggesting an essential role of semi-ring porous wood in TMCF trees to acclimate to actual climatic variations^33^. Violin plot results confirm the influence of climate on functional anatomical traits and the broadly defined effect of drought events on the variations in vessel traits. During specific Peruvian Andean drought events, *Cedrela* species developed narrow tree-rings and high vessel plastic variations that were linked to drier conditions, which increased hydraulic safety. Likewise, wider vessels were associated with moisture conditions, thus increasing hydraulic efficiency^34^, which was also detected in the *Cedrela* species during non-drought years. Differences in wood anatomical features, detected during drought and non-drought events, played an essential role in hydraulic adjusting and adaptation of the vessel traits to moisture deficit conditions^23^. Fonti et al.^35^, Rita et al.^23^ and Rodríguez-Ramírez et al.^15^, demonstrated the utility of vessel trait variations to detect an acclimation capacity, cavitation-resistant xylem in response to environmental factors such as drought events.

Although increasing vessel diameter implies a higher vessel vulnerability to drought^35^, specific vessel traits (*D*, V_*D*_, VI and D_*H*_) in *Cedrela fissilis* and *C*. *nebulosa*, showed poor hydraulic adjustment. This evidence suggests that both *Cedrela* species are more susceptible to drought events and therefore take on an increased risk of cavitation and/or embolism if drought conditions persist. On the other hand, *Cedrela angustifolia* displayed greater values of *D*, VI and D_*H*_; however, the low vessel density values found, which may contribute to adjusting the trade-off between the water supply system and wood anatomical features, may be part of the resilience strategy to drier site conditions^30^. Although, the risk of hydraulic failure would increase dramatically under extreme drought episodes^21,36^, specifically if vessel characteristics (*D*, VI, D_*H*_ and V_*D*_) are dysfunctional. This is consistent with the decrease in porosity at the intraspecific level reported under drier conditions^37^.

The vessel trait during DYs and NDYs in *Cedrela* species are indicative of the adaptive mechanisms to climatic events. Our study found that the vessel trait responses under specific drought intensities (moderate, severe and extreme) displayed a large variability among *Cedrela* species thereby avoiding the blockage of the hydraulic pathway^38^. Wider vessels may therefore be an adaptive strategy in high moisture environments in so far, a higher hydraulic capacity enables rapid adjusting^39^. This marked relationship tends to track specific climatic variations in times of climate change and implies retention of ancestral ecological features (phylogenetic niche conservatism^40^). The ability of trees to adjust the wood anatomical characteristics of vessel trait (hydraulic diameter, length, vessel density)^10,41^ and can provide information about climate effects. Therefore, the hypothesis that the functional vessel traits^36^, including growth-ring width, might be affected by specific drought events was supported. As drought effects on *D*, VI, D_*H*_ and V_*D*_ were linked to specific climatic signals (Figs. 5, 6), we demonstrated the ability to distinguish the vessel trait variations between drought and non-drought for *Cedrela* species and specific drought intensities of the *Cedrela* species. Even though each of the *Cedrela* species responded differently to climatic oscillations, this could be observed in the vessel traits that showed a reduction during drought years, suggesting a greater restriction in hydric-deficit^42^, in comparison with those vessel traits developed during non-drought years.

Despite the long-standing recognition of the importance of climate variation on tree ring assessment for the TMCF tree species^43^, the drought influence assessment on vessel trait is relatively new. Climate and environmental effects on vessel traits trigger resilience species may help understand how TMCF tree species will respond to further climate change^20^. We found that T_max_ and EvT influenced specific vessel traits (e.g., vessel diameter, vulnerability index) developing narrow vessels), hence functional adaptive in xylem properties^40^ as other TMCF tree species^15^, however, a high number of narrow vessels is significantly safer in terms of embolism resilience compared to a few large vessels^44^. Likewise, the vulnerability index (VI) was influenced by T_max_ and EvT only in *Cedrela fissilis* (Fig. 7) because of the species occur in high elevations (2588 m a.s.l). This could be explained by the resilience capability to specific drought years, providing xeric adaptation^36^. Notwithstanding, these results should be taken with caution since we did not account for the total range population distribution of each Peruvian Andean *Cedrela* species. TMCF community structure differences could be a result of environmental features, influencing the wood anatomical plasticity adaptation to drought periods.

Our results confirm the vessel traits trade-off for *Cedrela* species is in line with other intra-species studies which have assessed the effect of drought on TMCFs^20,45^. These wood anatomical variations are not usually recorded in growth ring-width and can provide a wider range of climatic response data to better interpret drought-adaptive capacity. Our vessel traits analysis has provided a new step forward for a broader understanding of the Peruvian Andean *Cedrela* species' response to drought events. Dendro-wood anatomy tools could be used as proxies in climatic studies assessing ecosystem response to climatic oscillations. There is a clear need to explain how montane tree species adjust their vessel architecture to optimal growth with minimal costs related to their hydric use^42^. An integration between dendroecological and wood-anatomical traits can help to illuminate the effect of climate-local adaptation of TMCF tree species. These responses help elucidate when and where, within a species’ range, vulnerability to drought-stress might be expected, and how much plasticity can buffer a rapidly changing environment due to climate change as exemplified by TMCFs.

## Methods

### Study sites

This study was conducted at three sites from Peruvian TMCFs (Supplementary Fig. S1A). The study sites possess a subtropical montane climate with a dry winter (Cwb^46^). Soils are mainly Leptosol to Andisol-humic (Th) and Leptosol-Cambisol-Regosol^47^. The forests have been subject to moderate anthropic intervention (e.g., illegal logging and familiar agriculture) and are affected in small areas by landslide dynamics.

### Sample collection and processing

We sampled 21 *Cedrela fissilis* trees from the Mamac locality; 17 *C. nebulosa* trees from the Agua de las Nieves locality; and 11 *C. angustifolia* trees from the Salinas de Alcanfor locality (Fig. S1B). *Cedrela* species with a diameter at breast height ≥ 40 cm were selected from each site to be cored twice at ~ 1.3 m (breast height) with a borer of 5 mm inner diameter (Häglof^®^, Langsele, Sweden)^48^. The increment cores were dried at air-room temperature, glued onto wooden supports, polished with sandpapers of decreasing grit size (from 40 to 1500), and prepared for optimal visualization of tree rings and vessel structure in cross section^49^. Wood dust inside the vessel lumina was removed with a compressed air device^20^. We deposited the dried specimens in the Selva Central Oxapampa Herbaria-(HOXA), Missouri Botanical Garden, Oxapampa, Pasco, Peru. Wood core preservation samples were preserved according to Speer^22^ and deposited at Dendrochronology Lab, Universidad Continental, Huancayo, Peru.

### Tree ring chronology development

We measured ring widths using a stereoscopic microscope, with the MeasureJ2X program and a Velmex tree-ring measuring system (Velmex, Inc., Bloomfield, NY, USA) with 0.001 mm accuracy. Tree-ring series were cross-dated using the Southern Hemisphere criteria, i.e., assigning to every ring the year in which growth started^50^. Cross-dating quality was statistically verified with the software COFECHA^51^ which computes correlation coefficients between overlapping segments of each tree-ring series and a mean reference series that allow us to identify cross-dating errors, false rings, and missing rings^52^. To obtain the average of detrended tree-ring width indices (RWI), we standardized raw ring-width series using a negative exponential function or lineal regression with a negative slope^53^. We used this analysis because we had two site sample lengths (between 59 and 62 years), which were flexible enough to preserve the low frequency of short tree-ring series while maximizing the climate signal^52^. We performed the analysis using the RCSigFree software^54^. Non-climatic trends were removed from each tree-ring series using a cubic spline of 12 years flexible enough to preserve 50% of the variance over a wavelength of 12 years and emphasize the low-frequency variation for each time series^55^. The chronology's quality was assessed through the R-bar and EPS statistics. The R-bar represents the mean inter-series correlation coefficient for all possible pairings among tree-ring series from individual cores, which examine the intensity of the common signal in all chronologies through time^56^. EPS values between 0.70 and 0.85 contain valuable climatic information, which quantifies the common signal strength and quality present within each chronology54.

### Climate data

For each study site, we obtained climate data for mean maximum temperature (T_max_), mean minimum temperature (T_min_) in °C, and monthly precipitation (Prec) in mm from nearby weather stations (Comas, 11° 44′ S, 75° 7′ W; 3300 m a.s.l., for Mamac locality; and Huasahuasi, 11° 15′ S, 75° 37′ W; 2750 m a.s.l., for Agua de Nieve and Salinas de Alcanfor localities; Fig. S1C) with records spanning from 1960 to 2014 (Fig. S1C).

### Climate and tree-growth relationships

To assess the climate sensitivity of *Cedrela* growth, we used Pearson’s correlation coefficients as a measure of similarity using SigmaStat v.4 (Systat software, Jandel Scientific, California, CA, USA). They were calculated between the tree-ring chronologies and monthly climate data (T_max_, T_min_ and Prec) of the previous-year January until the current-year June.

### Historical drought events over Peruvian Tropical Andean cloud forests

Recent severe droughts in the Peruvian TMCFs were linked with warm sea surface temperature (SST) anomalies over the tropical Pacific (SP) and tropical Atlantic developing a decrease in precipitation^57^. Consequently, drought events can be distinguished by multiple dimensions, that include their severity, duration, and frequency. In our study. We assessed the effect of drought on vessel traits, selecting 13 specific historical drought years that affected the tropical rainforest region in Peruvian Amazonia according to Jimenez et al.^58^ and Gloor et al.^59^. The drought events were classified into three intensities influenced by the SST anomalies: (1) moderate; ranging from 1.65 of 1.96 (1988 and 2004 years); (2) severe with rang from 1.96 to 2.58 (2005 and 2015, 2016); and (3) extreme having values > 2.58 (1982, 1983, 1990, 1994, 1997, 1998, 2010 and 2014)^58^. Subsequently, we identified the drought years for each exactly dated tree-ring chronology in order to recognize those rings in the digital images (Fig. 1D). When the drought events were consecutive, the information on the vessel traits were considered as independent.

### Drought events effect on tree growth

We performed a Superposed Epoch Analysis (SEA^60^) to explore the effect of the 13 specific historical drought years on RWI from the three *Cedrela* species. SEA links RWI time series with a list of drought events^59^. For each drought event, as 7-year window was considered which included 3-year before and 3-year following the event. The 7-year windows for all the events were superimposed and averaged to obtain the mean pattern of RWI related to drought events. The mean RWI pattern for the selected years was statistically evaluated for significance (95% confidence interval) by performing 1000 bootstrap simulations^60^ using random years from the RWI record. The analyses were performed using the R-program with *dplr*-package^61^.

Next, to determine the drought effects on RWI for each *Cedrela* species, we selected specific historical drought years (i.e., 1983, 1988, 1994, 1998, 2005, 2010, and 2014) and, we assessed three sensitivity indicators: resistance (*Rt*; Eq. 1), recovery (*Rc*; Eq. 2) and resilience (*Rs*; Eq. 3) based on criteria of Lloret et al.^62^ as:

1 $$Resistance \left(Rt\right)=\frac{{Ring\,\, width}_{t}}{{Ring \,\,width}_{t-2}},$$

2 $$Recovery \left(Rc\right)=\frac{{Ring \,\,width}_{t+2}}{{Ring \,\,width}_{t}},$$

3 $$Resilience \left(Rs\right)=\frac{{Ring \,\,width}_{t+2}}{{Ring \,\,width}_{t-2}},$$

where Ring width *t* is the growth width of the annual ring during the corresponding year *t*, Ring width t-2 is the average ring width for the 2-years preceding the year *t* and Ring width *t* + 2 is the average ring width for the 2-years following the year *t*^62^. We used *post-hoc* Tukey’s test to compare the mean between each pairwise combination of groups. We performed the analyses using BoxPlotR: a web-tool for the generation of box plots (http://shiny.chemgrid.org/boxplotr/). We did not consider successive drought periods such as 1982, 1997, 2005, 2015–2016 to avoid any overlaps with other low-growth periods^62^.

### Digitalization of growth-rings and xylem vessel traits

*Cedrela* wood is mainly characterized by ring-porous, or semi-ring porous with narrow vessels randomly scattered throughout the tree ring^63,64^. The vessels in the earlywood are distinctly larger than those in the latewood of the previous tree ring. To perform vessel analyses, we selected the eight cores that best fit the chronology for each *Cedrela* species. From those, we assessed the correlation values of each tree-ring series against the master chronology at each site using the software COFECHA. We took a digital image of the selected wood cores using a high-resolution scanner (∼ 2400 DPI) and saved them in tiff format. For each digital area selected, we identified the growth ring area (defined by a parenchyma band) corresponding to the non-drought years (an average of 1 mm × 0.5 mm length) and the drought years (an average of 0.6 mm × 0.5 mm length) and we manually quantified and measured all the vessel traits using the software ImageJ (https://ij.imjoy.io/#; accessed on 20 June 2022^65^) (Fig. S2). For the analysis of two consecutive drought years (i.e., 1982–1983; 1997–1998; 2004–2005; and 2014–2016), we considered them independent measurements.

### Vessel traits measurements

To assess how Andean Peruvian historical drought years influenced the xylem hydraulic architecture, we considered four-vessel traits measures: vessel tangential diameter (*D*, μm), vessel density (V_*D*_, mm^−2^), vulnerability index (VI; range from 1 to 3^21^), and hydraulic diameter (D_*H*_, μm), (Table 1). These wood anatomical features serve as rough indicators of the plant’s ability to withstand drought-induced cavitation^36^. The vessel traits were quantified and manually measured for those tree-rings developed during specific drought years as well as for the two consecutive non-drought years before and after drought events at the three *Cedrela* species using ImageJ-Fiji4^65^. We measured a total of 8129 vessels (1340 for *Cedrela fissilis*, 2438 for *C*. *nebulosa* and 4351 for *C*. *angustifolia*) from the digital images.

**Table 1:** Overview of xylem vessel traits, their acronyms, units, description regarding the calculation methods, and references for the trait concepts.

Vessel trait	Acronym	Unity	Equation or measurement	Equation description	Function	References
Vessel diameter	*D*	μm	$D=\frac{P}{\pi }$	*D* = radial diameter of the circle having the same area as the measured xylem vessel by taking the perimeter *P* of each conduit	Assess how prevent embolism formation because of drought stress	Scholz et al.^36^
Vessel density	V_*D*_	mm^−2^	Quantified as the average number of conduits per 1 mm^2^		Could give a closer proxy for the diffuse-porous wood's hydric resilience capacity	
Vulnerability index	VI		$VI\frac{D}{{V}_{D}}$	VI values below 1.0 suggest a high degree of xeromorphy, while values above 3.0 would characterize mesomorphy. Calculated using the vessel diameter (*D*, μm) and the vessel's density (V_*D*_, mm^−2^)	Provides a rough estimate of the plant's capacity to tolerate the drought-induced cavitation	Carlquist^21^
Hydraulic diameter	D_*H*_	μm	${D}_{H}=\left(\frac{{\sum }_{n=1}^{N}{D}^{4}}{N}\right)$	Represents the mean diameter that all the vessels in a stem would have to correspond to the overall conductivity for the same numbers of conduits. Equivalent circle diameter *D* and reflects the hydraulic conductance of conduits	Is linked to fundamental environmental conditions and to maximize the climatic signals	Scholz et al.^36^; García-González et al.^37^; Souto-Herrero et al.^66^

To determine if the vessel trait values showed a significant difference between DYs and NDs for the *Cedrela* species, we standardized the values to the same scale to avoid bias in subsequent analyses. Subsequently, evaluated data normality (Wilcoxon–Mann–Whitney test) and homogeneity of the variance (c^2^ test). Next, we performed an Analysis of Variance (ANOVA) and Tukey's post-hoc multiple comparison tests. Finally, we assessed the three intensities of drought events (i.e., moderate, severe, and extreme) on the vessel traits for the three Peruvian *Cedrela* species. These analyses were performed in R-software using *vegan* and *ggplot2* packages^67,68^.

### Climate effect on vessel traits

To explore the climate effect on vessel traits, we performed a generalized linear mixed model (GLMM with Gaussian distribution^69^), to determine if specific climate variable as detected Rita et al.^14^ could influence vessel traits. We got climate data from the CHELSA v. 2.0 database (http://chelsa-climate.org/; accessed on 26 September 2021^70^) the layer resolution was c. 1 km2, with records spanning from 1980 to 2018. Each vessel traits of *Cedrela* (*D*; V_*D*_; VI; and D_*H*_) was used as response factors, T_max_, Prec and EvT as explicative factors and the individual as random factor. We selected each vessel trait from wood cross-section digital images for the recorded drought years (1982, 1983, 1988, 1990, 1994, 1997, 1998, 2004, 2005, 2010, 2014, 2015 and 2016), as well as for the two consecutive years before each drought event. We accessed the effects and importance of each explicative factors using multiple model inference, selecting the best model with the minimum Adjusted Akaike ´s information criteria (AICc^71^). We verified the data normality (Shapiro–Wilk test) and homogeneity of the variance (chi-square test), selecting the best fit model for response factors. We considered statistically significant variables with *P* < 0.05 and *P* < 0.01 values^72^. We performed all GLMM analyses using R-program with the *glm2*^73^, *lm4*^74^, *MuMIn*^75^and *ggplot2*^68^ packages.

## Supplementary Information

Supplementary Information.

## Abbreviation

TMCF: Tropical montane cloud forest
