# Supplementary material for: Plastic adjustments in xylem vessel traits to drought events in three Cedrela species from Peruvian Tropical Andean forests
Source: Sci Rep. 2022 Dec 7;12:21112. doi: 10.1038/s41598-022-25645-w (PMC9729185; doi:10.1038/s41598-022-25645-w)
Supplement: Supplementary file 1 — Supplementary Information. [file 41598_2022_25645_MOESM1_ESM.pdf]

# Supplementary information

## Plastic adjustments in xylem vessel traits to drought events in three *Cedrela* species from Peruvian Tropical Andean forests

Ernesto C. Rodríguez–Ramírez[1]*, M. Eugenia Ferrero[2], Ingrith Acevedo–Vega[1], Doris B. Crispin–DelaCruz[1,3], Ginette Ticse–Otarola[1] and Edilson J. Requena–Rojas[1]

[1]Laboratorio de Dendrocronología, Universidad Continental, Urbanización San Antonio, Avenida San Carlos 1980, Huancayo, Junín, Perú.

[2]Instituto Argentino de Nivología, Glaciología y Ciencias Ambientales (IANIGLA), CONICET, Avenida Ruiz Leal S/N, Mendoza, Argentina.

[3]Programa de Pós-Graduação em Ciências Florestais, Universidade Federal Rural de Pernambuco,Recife 52171-900, Brazil.

* Corresponding authors: Ernesto C. Rodríguez–Ramírez (erodriguezr@continental.edu.pe).

**Table S1.** Growth-ring statistics for *Cedrela* species at the three study sites in Peruvian Tropical Andean cloud forests.

| | *Cedrela fissilis* | *Cedrela nebulosa* | *Cedrela angustifolia* |
|---|---|---|---|
| Timespan | 1896-2015 | 1958-2016 | 1923-2016 |
| Series intercorrelation | 0.41 | 0.49 | 0.56 |
| Tree samples | 15 | 17 | 37 |
| EPS | 0.821 | 0.817 | 0. 810 |
| Rbar | 0.193 | 0.207 | 0.420 |

**Table S2.** GLMM with Gaussian distribution statistical analysis results between vessel traits and climate factors.

*Cedrela fissilis*

| Climate and environmental factors | **Vessel traits** | | | | | | | | | | | |
|---|---|---|---|---|---|---|---|---|---|---|---|---|
| | ***D*** | | | **V*D*** | | | **VI** | | | **D*H*** | | |
| | β | *P* | AICc | β | *P* | AICc | β | *P* | AICc | β | *P* | AICc |
| EvT | 0.005 | **0.018*** | | -0.004 | 0.324 | | 0.004 | **0.049*** | | -0.005 | **0.080**** | |
| $T_{max}$ | 0.545 | **0.040**** | 53.70 | 0.000 | 0.000 | 70.55 | -0.960 | **0.006*** | 44.70 | 0.000 | 0.000 | 69.50 |
| Prec | 0.000 | 0.000 | | -0.001 | 0.762 | | 0.000 | 0.000 | | 0.001 | 0.546 | |

*Cedrela nebulosa*

| Climate and environmental factors | **Vessel traits** | | | | | | | | | | | |
|---|---|---|---|---|---|---|---|---|---|---|---|---|
| | ***D*** | | | **V*D*** | | | **VI** | | | **D*H*** | | |
| | β | *P* | AICc | β | *P* | AICc | β | *P* | AICc | β | *P* | AICc |
| EvT | -0.002 | 0.047 | | -0.006 | **0.069**** | | 0.001 | 0.098 | | 0.000 | 0.000 | |
| $T_{max}$ | 0.837 | **0.017*** | 54.50 | 0.000 | 0.000 | 68.40 | 0.199 | 0.285 | 45.60 | -0.648 | 0.221 | 64.40 |
| Prec | 0.000 | 0.000 | | 0.098 | 0.367 | | 0.000 | 0.000 | | 0.001 | 0.804 | |

*Cedrela angustifolia*

| Climate and environmental factors | **Vessel traits** | | | | | | | | | | | |
|---|---|---|---|---|---|---|---|---|---|---|---|---|
| | ***D*** | | | **V*D*** | | | **VI** | | | **D*H*** | | |
| | β | *P* | AICc | β | *P* | AICc | β | *P* | AICc | β | *P* | AICc |
| EvT | -0.002 | 0.188 | | -0.006 | **0.069**** | | 0.001 | 0.143 | | 0.000 | 0.000 | |
| $T_{max}$ | 0.747 | **0.025*** | 44.75 | 0.000 | 0.000 | 69.78 | 0.120 | 0.345 | 36.50 | -0.538 | 0.189 | 64.40 |
| Prec | 0.000 | 0.000 | | 0.001 | 0.246 | | 0.000 | 0.000 | | 0.001 | 0.705 | |

*= $P < 0.01$; **= $P < 0.05$

*Study area and location of sampling sites*

1) Mamac locality, Mariscal Castilla District, Concepcion Province, Junin Department (11° 32′38′′S, 75°8′47′′W, 2588 m a.s.l.), average annual precipitation of ~925 mm and an average temperature of 10.1°C (Figure. S1B). The study site is located in southeast-facing steep slopes (35°) with a tree layer dominated by *Cedrela fissilis* Vell., Cinchona micrantha, *C. officinalis*, *C. pubescens*, *Ficus* spp.

2) Agua de las Nieves locality, Monobamba District, Jauja Province, Junin Department (11°18′28′′ S, 75°20′19′′ W, 2161 m a.s.l.), average annual precipitation of 925 mm and an average temperature of 10.1°C (Figure S1B). The study site is located in the east-west steep slope (15°) located near the creeks, the canopy stratum is composed of trees up to 25 m in height as *Cedrela nebulosa* T.D. Penn. & A. Daza and *Prumnopitys montana* (Humb. & Bonpl. ex Willd.) de Laub. The mid-canopy is mostly characterized by *Eugenia* sp., *Miconia* spp., *Myrcia* sp., among others.

3) Salinas de Alcanfor locality, Monobamba District, Jauja Province, Junin Department (11°18′15′′S, 75°20′58′′W, 1966 m a.s.l.), average annual precipitation of 520 mm and an average temperature of 13°C (Figure S1B). The study site is the east-west steep slope (20°), the canopy stratum is dominated by *Cedrela angustifolia* Sessé & Moc. ex DC., *P. montana*, *Juglans neotropica* Diels. The understory stratum is dominated by *Ficus* spp, *Eugenia* sp., *Miconia* spp. and *Myrcia* sp.

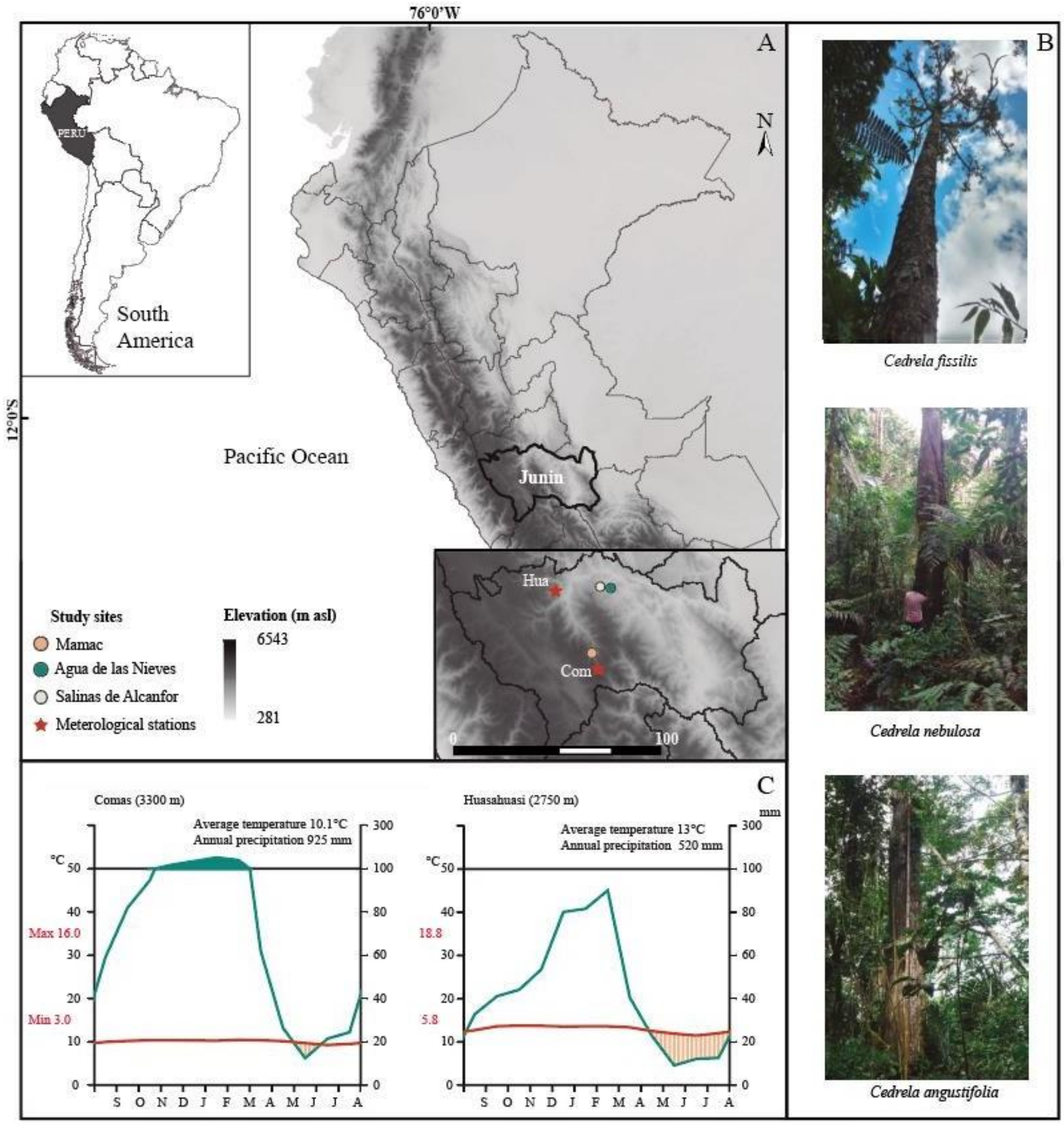


**Figure S1**. A) Map showing the location of the tree-ring sampling sites; B) View of *Cedrela* species; C) Walter climatic diagrams of Comas (period 1960–2002; -11°44′S, -75°7′W; 3300 m a.s.l.) and Huasuahuasi (period 1960–2002; -11°15′S, -75°37′W; 2750 m a.s.l.) weather stations. Green filled tips mean high moisture. This map was created by Ernesto C. Rodríguez-Ramírez using Adobe Illustrator v. 23.0 (www.adobe.com).

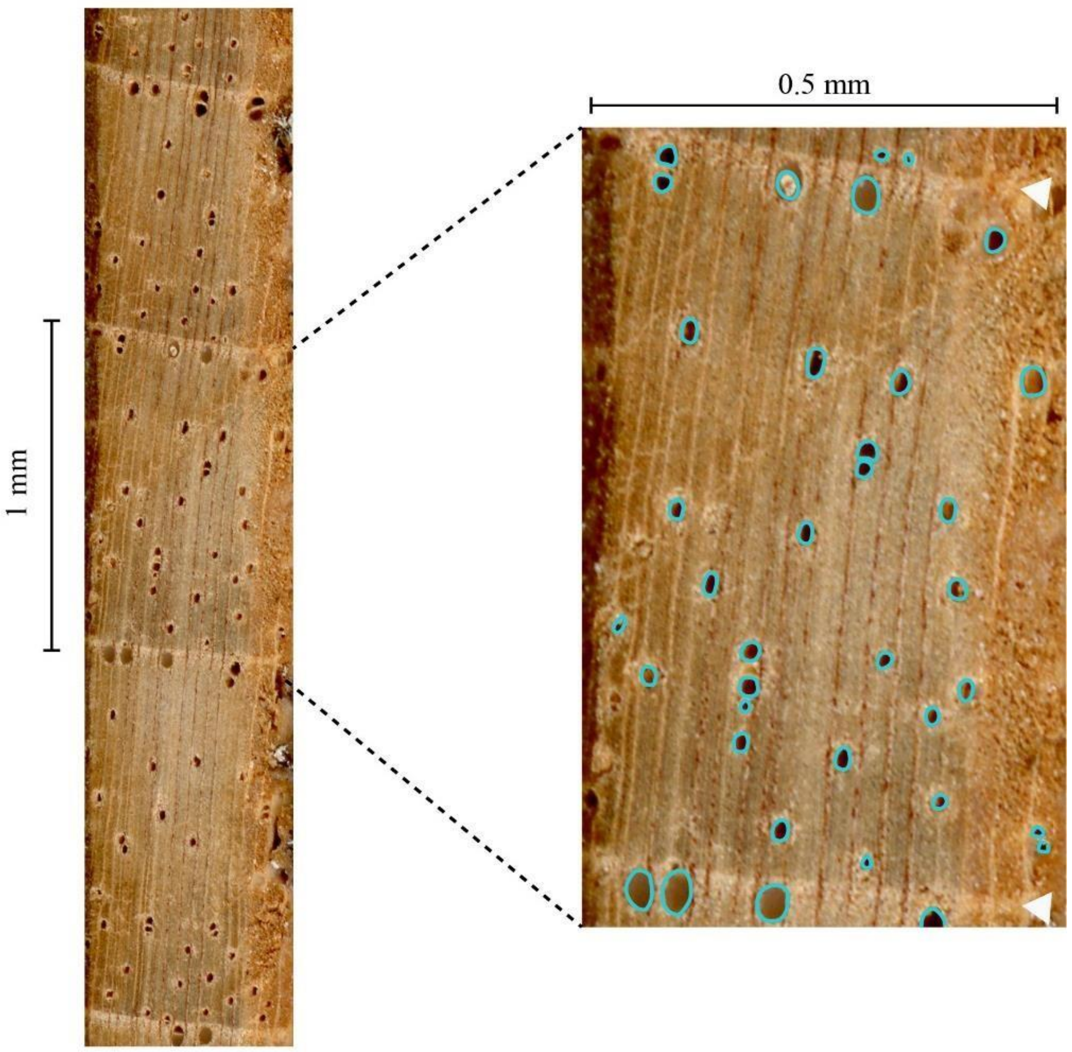


**Figure S2**. Assessment of xylem vessel traits (cyan color) in Peruvian *Cedrela* species as manually defined using the software ImageJ, in a region of ~1 mm of length × 0.5 mm of width of each tree ring. White arrows in the right panel show the location of the parenchyma wall of the grow-ring. This figure was developed by Ernesto C. Rodríguez-Ramírez using Adobe Illustrator v. 23.0 (www.adobe.com).
